# Playing the electric light orchestra—how electrical stimulation of visual cortex elucidates the neural basis of perception

**DOI:** 10.1098/rstb.2014.0206

**Published:** 2015-09-19

**Authors:** Nela Cicmil, Kristine Krug

**Affiliations:** Department of Physiology, Anatomy and Genetics, University of Oxford, Parks Road, Oxford OX1 3PT, UK

**Keywords:** visual cortex, electrical stimulation, perception, primate, optogenetics, decision-making

## Abstract

Vision research has the potential to reveal fundamental mechanisms underlying sensory experience. Causal experimental approaches, such as electrical microstimulation, provide a unique opportunity to test the direct contributions of visual cortical neurons to perception and behaviour. But in spite of their importance, causal methods constitute a minority of the experiments used to investigate the visual cortex to date. We reconsider the function and organization of visual cortex according to results obtained from stimulation techniques, with a special emphasis on electrical stimulation of small groups of cells in awake subjects who can report their visual experience. We compare findings from humans and monkeys, striate and extrastriate cortex, and superficial versus deep cortical layers, and identify a number of revealing gaps in the ‘causal map′ of visual cortex. Integrating results from different methods and species, we provide a critical overview of the ways in which causal approaches have been used to further our understanding of circuitry, plasticity and information integration in visual cortex. Electrical stimulation not only elucidates the contributions of different visual areas to perception, but also contributes to our understanding of neuronal mechanisms underlying memory, attention and decision-making.

## 1. Introduction

The visual cortex is perhaps the most thoroughly investigated of any brain system in mammals. In primates, visual cortex has been delineated into more than 30 distinct areas based on their anatomical and functional properties [1,2]. Research into visual cortical function reveals fundamental mechanisms underlying perceptual experience and also has the potential to improve our treatment of disorders such as amblyopia, blindness and visual hallucinations. Prominent methods currently used to investigate the function of visual cortex are often correlational and include neuroimaging, such as functional magnetic resonance imaging (fMRI), and electrophysiological techniques, such as single cell neurophysiology. fMRI is very useful for measuring changes in activity throughout the entire human brain that can be correlated with perception and behaviour. However, its spatial and temporal resolution is too low to reveal functional properties of individual neurons or small groups of cells, and it measures blood oxygenation level-dependent (BOLD) responses which are only indirectly coupled to neuronal firing [3–5]. In neurophysiological recording, an electrode is inserted into the brain to directly measure the firing of neurons, individually or in small groups [6]. This activity is correlated with simultaneous visual stimulation or perceptual reports to infer the information represented by neuronal firing.

It is often assumed that information represented in neuronal activity necessarily contributes to visual perception and informs behaviour. However, some neurons might echo information that is not used at downstream processing levels. One example is consistent neural tuning in visual cortex to anti-correlated binocular disparities that do not lead to a coherent visual depth percept [7,8]. In order to infer a direct contribution of the information represented by neuronal firing to perception, it is necessary to use causal experimental interventions. Demonstrating that direct interference in the firing patterns of the candidate neurons leads to a measureable change in the perceptual responses of the viewer is one of seven empirical criteria proposed to support a critical link between neurons and perception [9].

Causal experimental interventions for investigating the visual system include electrical microstimulation, lesion studies, transcranial magnetic stimulation (TMS) and transcranial direct current stimulation (tDCS). Electrical stimulation of visual cortex involves the introduction of electrical current into a small cortical region through an electrode placed on the cortical surface or a microelectrode inserted into cortical matter [10,11]. The current reversibly activates neurons in the vicinity of the electrode to fire action potentials [12]. The size of the activated region is affected by the type of electrode used and the strength of stimulation [13]. Lesion studies involve observing the effect of removing a particular brain area. These can provide a causal link between cortical areas and specific visual functions, but are irreversible and results can vary between individuals and change over time. TMS and tDCS are non-invasive approaches used to modulate neural activity, via electromagnetic induction from a coil placed on the scalp or electrical currents from scalp surface electrodes, respectively. TMS and tDCS have helped advance our understanding of sensorimotor function and multi-sensory integration, also involving vision (for a review see Yau *et al*. [14] ). However, electrical stimulation approaches have proved to be the most powerful tool for establishing a direct contribution of neuronal activity at different levels of visual processing to visual perception and cognition in a reversible and controlled way.

General principles of neural mechanisms for cognition and behaviour as revealed by causal approaches have been comprehensively reviewed elsewhere [15,16]. In this article, we consider the perceptual functions of visual cortex in primates as revealed by causal intervention methods with a special emphasis on the direct activation of small groups of visual cortical neurons. We explore a number of themes that become apparent when comparing results from human and non-human primates, striate and extrastriate cortex, and superficial versus deep cortical layers. We discuss why it works to ‘mix’ visual and electrical stimulation in cortex and what a significant number of unexplained gaps in the ‘causal map’ of visual cortex tells us about visual processing and perception. This leads to new questions and insights about the interaction between visual cortical activity, causal experimental approaches and perception.

## 2. Humans and monkeys detect electrical stimulation of visual cortex

Different visual cortical areas have been investigated with electrical stimulation by measuring the threshold current required for stimulation detection or by documenting the nature of the percept evoked by supra-threshold stimulation. These studies apply electrical stimulation without any specific simultaneous visual input.

### (a) Electrical stimulation of visual cortex in humans

Electrical stimulation of visual cortex in humans has the potential to reveal the distinct functional contribution of specific cortical regions to visual perception since humans can report their induced perceptual experiences. Since the surgery necessary for direct electrical stimulation comes with risk of infection and cortical damage, it is unethical to perform stimulation experiments on typical human volunteers. Neurological patients who already undergo surgical treatment for electrode placement, e.g. for localization of epileptic foci or for tumour resection, constitute an important potential participant group for cortical stimulation. For example, temporal lobe epilepsy is a common form of localized epilepsy [17], and as a result extrastriate visual areas located in the temporal lobe have been accessed for stimulation experiments [10,18–20]. Another important potential participant group are visually impaired individuals who volunteer to test electrical visual prostheses implanted in cortex, usually in area V1 and neighbouring regions [21–25]. Notwithstanding possible differences in activity between typical, healthy brains and those with neuropathology, patients' reports of their experiences under stimulation have revealed perceptual effects of activating particular groups of neurons in visual cortex.

#### (i) Electrical stimulation of area V1 in humans

**Figure 1.**
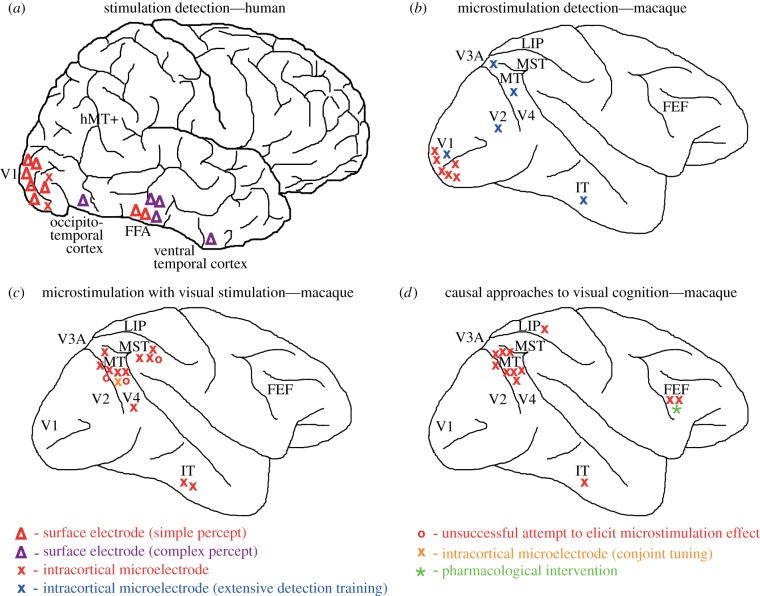
Overview of sites where causal stimulation experiments have been performed in the visual cortex (and selected connected areas) of humans and monkeys (see also tables 1 and 2). Sites are shown on schematic human and macaque brains, and indicate the visual cortical areas involved (not exact electrode positions). (*a*) Visual cortical sites of electrical stimulation in human patients where either a simple phosphene percept was evoked with a cortical surface electrode (red triangle) or with an intracortical microelectrode (red cross), or where a complex form percept was evoked (purple triangle—surface electrodes only). Most sites where larger currents evoke reportable percepts are around primary visual cortex (V1) and the fusiform face area (FFA). (*b*) Visual cortical sites for which macaque monkeys have detected intracortical electrical microstimulation either without (red cross) or with extensive training to specifically detect electrical microstimulation (blue cross). For extrastriate visual cortex, specific detection training appears to be required. (*c*) Visual cortical sites in macaque monkeys, where low current, intracortical electrical microstimulation was combined with simultaneous visual stimulation. Experiments successfully (red cross) or unsuccessfully (red circle) biased animals' perceptual decisions towards the neuronal tuning preference of the stimulated site. In one experiment, microstimulation biased perceptual decisions towards the conjoint neuronal tuning for two visual parameters (orange cross). (*d*) This figure summarizes the cortical sites discussed in this review, where causal approaches were used to investigate visual cognition, including working memory, attentional and decision-making processes, with intracortical electrical microstimulation (red cross) or pharmacological intervention (green star). IT, inferotemporal area; LIP, lateral intraparietal area; FEF, frontal eye fields; MST, medial superior temporal area; MT, middle temporal area.

Cortical surface stimulation of the human occipital pole in the region of the calcarine fissure, the location of area V1 and other early visual areas, results in the sensation of light, called a phosphene [21–28], described as ‘like a star in the sky’ [21] (figure 1*a*). Locations of phosphenes with respect to the stimulating electrode agree with retinotopic maps of the visual field in cortex, and patients report that phosphenes move in the direction of voluntary eye movements, demonstrating the retinocentric representation of space in early visual areas [21]. Phosphenes are reported to be around 1 mm in diameter and can be elicited by current levels between 1 and 5 mA [21,22,25,26] (methodological parameters are summarized in table 1). Substantial supra-threshold stimulation sometimes produces a second phosphene that follows a mapping pattern reflected about the horizontal meridian, the horizontal midline across the visual field [21,26]. One interpretation of this effect is that higher currents can spread to neighbouring regions of cortex, for example, from dorsal to ventral V1 [39–41]. Overall, the electrical stimulation of area V1 reliably induces perception of retinotopically organized simple light sensations.

**Table 1.** Summary of electrical stimulation detection studies in humans and non-human primates. This table compares the methods employed in studies of the detection of electrical stimulation in visual cortex discussed in this review. Abbreviations used: NHP, non-human primate; VEP, visually evoked potential; ERP, event-related potential; fMRI, functional magnetic resonance imaging; LFP, local field potential; RF, receptive field; Pt/Ir, platinum-iridium; MU, multi-unit neural activity; IT, inferotemporal cortex. (−/+) represents negative- or anode-leading biphasic stimulation, (+/−) represents positive- or cathode-leading biphasic stimulation. For biphasic stimulation, stimulating current (voltage) is reported as the zero-to-peak amplitude and pulse duration is reported as the duration of each positive or negative phase.

study	neurological diagnosis	stimulation method	stimulating current, mA (*voltage*, *V*)	stimulation frequency, Hz	stimulation pattern	pulse duration, ms	recordings	cortical area
*human studies*	*** ***	*** ***	*** ***	*** ***	*** ***	*** ***	*** ***	*** ***
Penfield [10]	epilepsy	surface electrode	(*1–5*)	40–100	square-wave	2–5	background activity and seizures	temporal lobe and central sulcus
Brindley & Lewin [21]	blindness	surface electrode	(*8–56*)	30 (range 25–4000)	train of short pulses	0.03 (range 0.01–1)	no	occipital pole
Dobelle & Mladejovsky [26]	occipital surgeries^a^	surface electrode	1–12	12–200	monophasic and biphasic	0.06–2.0	no	occipital cortex
Dobelle *et al*. [22]	blindness	surface electrode	0.5–3	50	biphasic (−/+)	0.25	no	medial occipital cortex
Bak *et al*. [27]	epilepsy	intracortical microelectrode	0.01–2	100	biphasic (−/+) and (+/−)	0.2	no	occipital pole
Allison *et al*. [18]	epilepsy	surface electrode	2–10	50	bipolar of adjacent electrodes	0.1	VEP to colour adaptation	occipito-temporal cortex
Schmidt *et al*. [23]	blindness	intracortical microelectrode	0.001–0.04	75–200	biphasic (−/+) and (+/−)	0.2–0.8	no	occipital cortex
Puce *et al*. [19]	epilepsy	surface electrode	2–10	50	bipolar constant-current	0.2	face-specific ERP (N200)	occipito-temporal cortex
Dobelle [24]	blindness	surface electrode	(*10–20*)	30	biphasic (−/+)	0.5	no	occipital cortex, mainly V1
Lee *et al*. [29]	epilepsy	surface electrode	1–15	50	biphasic (+/−)	0.3	no	occipital and temporal cortex
Pollen [25] (after Pollen [30])	hemianopsia	surface electrode	0.8–1.5	20, 30, 60 and 120	biphasic (+/−)	0.25	no	V1
Murphey *et al*. [20]	partial seizures	surface electrode	1–4	200	biphasic	0.2	fMRI and LFP of colour responses	ventral temporal cortex
Murphey *et al*. [28]	epilepsy	surface electrode	∼0.4–7	200	biphasic	0.2	fMRI and LFP for RF mapping, and functional localization	occipital and temporal cortex
study	species	stimulation method^b^	stimulating current, µA	stimulation frequency, Hz	stimulation pattern	pulse duration, ms	recordings	cortical area
*monkey studies*	*** ***	*** ***	*** ***	*** ***	*** ***	*** ***	*** ***	*** ***
Doty [11]	macaque	platinum-plated electrodes	10–1000	2, 50 or 100	rectangular constant current	0.2–1.0	no	multiple
Bartlett & Doty [31]	macaque	microelectrodes	2–250	50	cathodal and anodal pulses	0.2	background MU activity	V1, layers V-VI
Bartlett *et al*. [32]	macaque	Pt/Ir macro- and micro- electrodes	10–1500	10, 50 or 100	cathodal and anodal pulses	0.2	no	V1
DeYoe *et al*. [33]	macaque	Pt/Ir microelectrodes	1–25	50 or 100	cathodal pulses	0.2	background MU and response to flashes	foveal and perifoveal V1
Murphey & Maunsell [34]	macaque	Pt/Ir microelectrode	1–30	200	biphasic	0.2	MU response properties, and RF location and size	V1, V2, V3A, V5/MT, and IT
Tehovnik & Slocum [35]	macaque	Pt/Ir microelectrode	1–30	200	biphasic (+/−)	0.2	MU RF location	V1
Ni & Maunsell [36]	macaque	Pt/Ir microelectrode	1–50	200	biphasic (−/+)	0.2	MU RF location	V1
Schiller *et al*. [37]	macaque	Pt/Ir microelectrode	15–160	200	biphasic	0.2	MU RF location	V1
Ghose & Maunsell [38]	macaque	Pt/Ir Utah microelectrode arrays	1–25	200	biphasic (+/−)	0.2	MU RF location	V1

^a^In the study of Dobelle & Mladejovsky [26], occipital surgical resections were carried out for a variety of reasons, including tumours, arteriovenous malformation and benign cysts.

^b^All monkey detection studies used intracortical electrodes for brain stimulation.

Descriptions of perceived phosphenes, however, are not uniform across volunteers. In most cases, phosphenes were round, but occasionally patients have reported elongated phosphenes ‘like half a matchstick at arm's length’ [21]. Some studies reported a lack of colour sensation upon stimulation [21,29], while in other cases the chromatic effects of phosphenes were vivid reds, blues or greens or ‘unreal’ colours, described as being ‘from another world’ [26]. Some of these differences could be due to on-going cortical changes after deprivation of sight in blind patients, or differences in electrode placement, stimulation patterns or local cortical circuitry.

An alternative to electrical stimulation with electrodes placed on the cortical surface are stimulation microelectrodes inserted into the cortical matter—allowing finer control of the precise location of the current injection. When such ‘microstimulation’ of the occipital pole was applied through intracortical microelectrodes, reported phosphene sensations were very similar to those elicited with surface electrodes: small, light, simple forms, which were either whitish-yellow or brightly coloured [23,27]. Current thresholds required to produce phosphenes by intracortical microstimulation are 10–100 times lower than those required for non-penetrating surface stimulation (methodological parameters are summarized in table 1); stable detection thresholds can be reached at below 50 µA [23,24].

#### (ii) Electrical stimulation of extrastriate visual cortex in humans

In contrast to area V1 and its immediately neighbouring regions near the occipital pole, it is generally more difficult to evoke detectable sensations with electrical stimulation of extrastriate visual areas using surface electrodes [28,29,42]. Even when detectable sensations are elicited, reports differ regarding the content of the evoked sensation. In some studies, patients reported sensations of ‘complex forms’, such as faces or visual scenes from memory [10,19,29], while in other studies only simple form sensations, such as phosphenes or colour spots, were evoked [18,20,28] (figure 1*a*). The circuitry of visual areas further downstream may generally support more complex electrical activity patterns that cannot be readily induced by focal electrical stimulation. We discuss in §2*b*(i) how these differences in evoked percept might arise from anatomical and functional differences between primary and extrastriate visual cortex in both the human and non-human primate brain.

In cases where a detectable sensation could be elicited from stimulation of higher visual areas in humans, the current threshold for detection at that site is similar to thresholds found in early visual areas, suggesting that there might be particular regions of extrastriate visual cortex that naturally support more focal activation patterns similar to those induced by electrical stimulation [28]. Earlier studies of human extrastriate cortical stimulation, which include reports of sensations of complex forms, generally used lower stimulation frequencies (50 Hz) than more recent studies that reported only simple phosphenes (200 Hz) (table 1). Further research is required to understand how different electrical stimulation patterns might potentially lead to different percepts.

Crucial to linking cortical processing to perception, in some studies, neuronal response properties were characterized at the stimulated cortical site, and shown to relate to the evoked perceptual sensation. Allison *et al.* [18] recorded visually evoked potentials (VEPs) from chronically implanted cortical surface electrodes placed over occipital and temporal cortex of patients with epilepsy [18]. They found a significant colour adaptation-related VEP response over the lateral lingual and fusiform gyri, which upon electrical stimulation sometimes evoked colour sensations. Nearby regions, such as the medial lingual or cuneate gyri, did not show significant VEP adaptation responses to colour, and electrical stimulation evoked only monochrome phosphenes. Similarly, Murphey *et al.* [20] stimulated an electrode placed over the anterior colour centre (putative area V4α, mesial fusiform gyrus), localized with BOLD fMRI, of a patient with epilepsy [20]. The patient reported that electrical stimulation evoked a percept of a ‘blue, purple colour’, and subsequent local field potentials recorded with the same electrode showed the greatest response to blue-purple colour. This demonstrates a close link between selectivity for visual stimuli and contribution to colour perception of small sections of ventral temporal cortex.

It has not been possible, however, to demonstrate such tight links across multiple experiments in all cases. One investigation of face-selective regions in ventral temporal cortex, identified by face-specific N200 event-related potentials, showed that upon stimulation two-thirds of such sites either evoked face-related hallucinations or transiently disrupted patients' ability to name familiar faces [19]. But in another study, stimulation of an electrode placed over the fusiform face area (FFA) in ventral temporal cortex either failed to produce a percept when stimulated, or evoked only a simple phosphene [28]. These differing results may be due to individual differences in extrastriate function between patients. But, they might also reveal current limitations in our understanding and control of the effects of direct electrical stimulation on the volume of brain tissue below a cortical surface electrode [43], particularly in the absence of intracortical microelectrode data from extrastriate visual cortex (figure 1*a*), which can specifically activate smaller groups of neurons and therefore provide more control.

Since causal stimulation approaches require specific, rare patient populations, the time available to test human volunteers is dictated by clinical demands and is therefore limited. It is often not possible to perform fully controlled psychophysical studies on multiple volunteers (but see [23,24]). Brain regions available for testing are limited by the type of neuropathology and the surgical access that is indicated for the particular patient. For example, apart from in early cortex-wide stimulation experiments [42], dorsal visual areas such as V3A and hMT+ have not been specifically investigated using electrical stimulation methods in humans. Therefore, there is a ‘gap’ in the causal map of human visual cortex (figure 1*a*). Under the ‘dual stream hypothesis’ of vision, dorsal visual stream areas are concerned with vision for control of movement and visual motion perception [44,45]. However, in the only electrical stimulation study in which patients reported moving phosphenes, the visual areas involved were mainly medial or ventral, rather than dorsal [29]. It is also possible that in that study the perceived motion was due to eye movements, which were not measured (eye movement recordings would help to interpret human visual cortical stimulation studies more generally). Therefore, electrical stimulation studies in humans have to date provided little evidence for the dual stream hypothesis.

Overall, electrically stimulating early visual cortex in humans elicits reliably simple visual phosphenes in the predicted retinotopic location, but evidence for complex visual percepts from electrical stimulation is limited. While significant practical and ethical constraints provide challenges for such experimentations, crucial experiments remain to be done to reveal how cortical signals give rise to specific sensory experiences.

### (b) Electrical stimulation of visual cortex in non-human primates

Owing to the inherent limitations of studies with human patient volunteers, non-human primates have also been used in electrical stimulation detection studies. In detection tasks, animals report the presence or absence of electrical stimulation within a given time period, for example, by pressing a lever [11] or making an eye movement (saccade) to an appropriate target [34]. Intracortical microelectrodes, rarely used in human studies, can be used with animal models. Early studies that stimulated sites throughout cortex used currents of up to 1 mA, while more recent studies that focus on the primary visual cortex (V1) in well-trained animals tend to stimulate within a much lower range of 1 to 50 µA (see table 1 for a summary of methodological parameters).

#### (i) Detection of electrical stimulation of visual cortex in non-human primates

With little prior training to recognize electrical stimulation, rhesus monkeys can reliably detect strong electrical stimulation of area V1 [31] (see also [15]). However, extensive training, numbering over thousands of trials, is necessary to achieve stable low detection thresholds (i.e. below 50 µA) at V1 sites and to reliably detect electrical microstimulation in extrastriate areas [31,34,36] (figure 1*b*). This resembles the pattern found in humans, described above.

**Figure 2.**
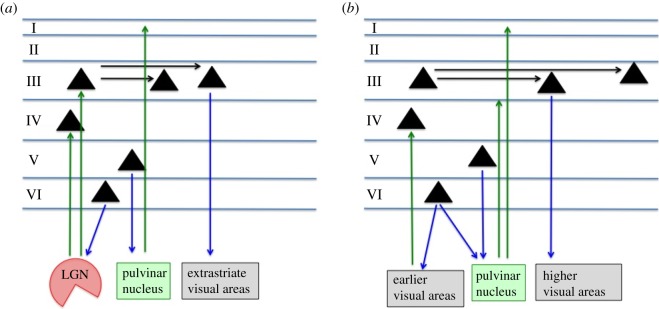
Overview of some important layer-specific connections for (*a*) primary visual cortex and (*b*) extrastriate visual cortex. Differences in these connections may underlie the differential effectiveness of electrical microstimulation between visual cortical areas, and for different layers within primary visual cortex, without extensive prior detection training. Layer V and VI projections form part of the fast reciprocal connections between primary visual cortex and the lateral geniculate nucleus (LGN) and pulvinar [47,50–52]. This may explain why lowest detection threshold currents are found in these deep layers [27,31,34,35]. Moreover, horizontal connectivity links spatially closer clusters of neurons in primary visual cortex [46–49], while horizontally connected clusters of cells in extrastriate areas can be spaced more widely, up to 8–10 mm away [53–55]. Detection of electrical stimulation may be more reliable in primary visual cortex (without extensive prior training) because stimulation activates axons connecting nearby neuronal clusters serving similar parts of the visual field. Major inputs to visual cortex are depicted in green, output projections in blue and intrinsic connectivity in black. A significant part, especially of the intrinsic cortical connectivity, was omitted from these schemata for clarity.

In both humans and monkeys, such differences in detectability of electrical stimulation in primary versus extrastriate visual cortex are likely to be related to differences in anatomical and functional connectivity. In the rhesus macaque, extrastriate visual areas show a more extended pattern of intrinsic horizontal connectivity. Although individual axons in area V1 can be up to 8 mm long, clusters of monosynaptically connected cells tend to be less than 4.5 mm apart [46–49] (figure 2*a*). In extrastriate visual areas, however, directly connected clusters of neurons can be more than 10 mm apart and such clusters tend to be more widely spaced [53–55] (figure 2*b*). This pattern of local connections is important because it has been suggested that electrical microstimulation directly activates axons in a volume tens of micrometres in diameter [12]. The potentially increasing spatial range, coupled with a decreasing cortical magnification in many higher visual areas relative to V1, could result in a more widespread, less detectable microstimulation effect.

Regarding functional connectivity, area V1 may respond to natural visual input in a spatially and temporally restricted pattern, similar to that induced by artificial stimulation, perhaps reflecting its retinotopic, columnar organization with small receptive fields. Cortical regions higher in the visual processing hierarchy may, on the other hand, support neuronal activity patterns that are more spatially distributed and temporally complex—in other words, quite different from the pattern introduced by artificial stimulation. In the absence of appropriate simultaneous visual stimulation, focal electrical microstimulation of extrastriate cortex might not even lead to significant activation beyond the local area or might lead to widespread inhibition. Baseline firing rates of neurons could also affect how effectively they can be activated by electrical stimulation. For example, in studies with cats, it was found that surface electrical stimulation of regions with high spontaneous activity can result in the inhibition of neural responses, particularly with low current strengths [56]. Such functional differences may underlie whether or not artificial stimulation patterns can effectively propagate action potentials and thus support perception prior to specific detection training.

Experiments measuring detection thresholds for microstimulation applied to different cortical layers of macaque V1 have forged further links between anatomy and stimulation detectability [31,33–35,57]. Current thresholds generally decrease as a function of cortical depth and are lowest in the deepest layers (V and VI), in line with findings in humans [27]. The lower thresholds found for deep layers may depend upon the brain circuits to which neurons in these layers connect. In macaque V1, layers V and VI contain pyramidal cells that project to subcortical nuclei: projections from layer V neurons terminate in the pulvinar, superior colliculus and brainstem centres; layer VI neurons project to the lateral geniculate nucleus (LGN) [47,50] (figure 2*a*). Electrical stimulation of the LGN in the macaque is detectable at mean currents of 40 µA without specific detection training [51]. The direct fast feedback loop from V1 deep layers to the LGN and back [52] may therefore magnify the effect of V1 electrical stimulation in a spatially localized fashion via the sensitive LGN, and thus aid detectability. At strong currents, LGN stimulation can also activate the pulvinar, and vice versa [58], indicating another subcortical pathway that could transmit or even amplify the microstimulation signal to higher brain areas. The efficacy of focal electrical microstimulation may therefore be intrinsically linked to the neuronal projection pattern of different cortical layers.

#### (ii) Training animals to detect electrical stimulation

After extensive detection training, all visual cortical areas in which microstimulation detection has been tested (V1, V2, V3A, V5/MT and inferotemporal cortex, known as IT) have yielded reliable, low detection thresholds [34,36]. Post-training detection thresholds increase along the visual cortical hierarchy but overall remain relatively low, from around 6 µA in area V1 to 11 µA in area IT [34]. This suggests that neuronal signals of similar magnitude in any part of visual cortex can be accessible to behaviour after sufficient learning and practice.

Local cortical changes with microstimulation detection training appear to occur through a slow process, the time scale of which resembles that of perceptual learning of sensory stimuli [15,59]. Alternatively, electrical stimulation of extrastriate visual cortex might immediately affect perception, but for some reason, the animal does not report it. This could occur if the induced sensation does not appear task-relevant, for example, if it is qualitatively very different to the visual stimulus to which the animal was exposed during training. Improvement in detection accuracy over time may therefore reflect the animal gradually learning that the qualitatively different sensation is indeed behaviourally relevant for obtaining rewards in the detection task [57]. In any case, the behavioural data does not support a sudden realization that responses should be guided by a qualitatively novel sensation, which would be expected to result in a sudden performance improvement [15,34].

In order to investigate the mechanisms by which microstimulation detection thresholds improve, Ni & Maunsell [36] trained monkeys to detect low currents of stimulation of small (3 × 3 mm) V1 sites with a defined receptive field location [36]. After learning to detect currents of 6–10 µA, animals' detection thresholds for visual stimuli placed within the site's receptive field were reciprocally increased. The point has previously been raised that on-going microstimulation could damage neurons near the stimulation site [60]; this may be responsible for the subsequent changes in visual discrimination thresholds. However, after retraining on the visual task, animals' visual thresholds decreased back to baseline levels, while microstimulation detection thresholds reciprocally rose. The recovery of visual detection thresholds suggests that the original decrease was because of reversible synaptic changes [36]. Additionally, for the first V1 sites stimulated in each monkey, the number of trials at which the highest stimulating current (50 µA) was undetectable was greater than for subsequent sites, revealing a small general training effect across area V1 as a whole (discussed in [57]; see also [11]). These results indicate that V1 retains significant plasticity into adulthood, but there appear to be fundamental limitations on the range of different neural activation patterns accessible to behaviour that can be simultaneously supported by the cortical circuitry under a given training regime.

Earlier we saw that detection of artificial stimulation in V1 is different to other visual areas, precisely because detection of strong stimulation is reliable with no prior experience. Nevertheless, it would be interesting to explore how detection (or discrimination) thresholds for tailored visual stimuli versus an artificial microstimulation signal in extrastriate cortex might be reciprocally affected by training. This could provide further insight into the relationship between the patterns of neural activation evoked by microstimulation and by visual processing in these areas. If the difference between natural and focally induced artificial activation patterns were greater for extrastriate visual areas compared with V1, then we would expect learning to take longer and the relative reciprocal decrease in visual discrimination ability after stimulation detection training to be greater for extrastriate areas.

#### (iii) Limitations of stimulation detection experiments in animals

Stimulation detection experiments in non-human primates have provided important information about visual cortical function. However, as for human studies, response properties of the stimulated neurons have not been routinely characterized. In some cases, background multi-unit (MU) activity levels were recorded, while in others, only receptive field locations of stimulated sites were measured (see table 1 for details). Detailed information about the tuning properties of stimulation sites would be useful for further interpreting the results of some studies, for example, response properties of microstimulated V1 sites before and after microstimulation detection training in Ni & Maunsell [36]. A decrease in neuronal sensitivity to visual stimulation could explain the increase in visual detection thresholds; a subsequent increase in responsiveness after visual retraining could explain the regaining of sensitivity to visual stimuli. On the other hand, an absence of changes in neuronal responses would suggest that synaptic changes at other brain sites are responsible for the reciprocal detection threshold effect. For example, improved sensitivity to weak visual motion after perceptual learning correlates with changes in motion-driven responses of neurons in sensorimotor lateral intraparietal cortex (area LIP), but not in motion-selective visual cortical area V5/MT [61].

Another limitation of microstimulation detection studies is that animals usually report only the presence or absence of microstimulation and not the nature of any evoked percept. To our knowledge, only one study directly investigated the perceptual appearance of electrical microstimulation in monkey visual cortex [37]. Monkeys were first trained to compare two different visual targets and to saccade to either the higher contrast or the larger target. One of the visual targets was then replaced by electrical microstimulation of a V1 site with a corresponding receptive field location. Then the contrast and colour of the visual background was systematically changed until the targets evoked by microstimulation ‘disappeared’. Thus, it was revealed that microstimulation evoked the percept of a small spot that was darker than the display background and that ‘microstimulation spots’ were composed of a variety of low contrast colours [37]. However, these results are difficult to reconcile with human reports of bright, strongly coloured phosphenes during stimulation of V1 with intracortical microelectrodes [23,27] (see §2a). Overall, the contribution that the direct activation of particular cortical regions makes to the visual perceptual experience requires further investigation.

While we do not know enough about the qualitative nature of the artificially evoked sensory experience in monkeys, it is clear that animals can reliably detect electrical microstimulation with small currents in area V1 as well as in extrastriate visual areas, at least with appropriate training (figure 1*b*). The detection training studies in animals showed a reciprocal relationship between thresholds for visually and artificially evoked responses. This suggests that electrical activation of visual cortical neurons may produce a neuronal activity pattern quite different from that evoked by natural viewing of visual stimuli. This might be particularly evident in the context of viewing an unchanging empty background like in many detection studies. Therefore, we need to determine how the neural and visual context in which a cortical site is activated affects information integration and polysynaptic propagation to sensorimotor structures that initiate and control behaviour.

## 3. Electrical microstimulation ‘mixed’ with visual stimulation

One way artificial electrical activation of visual neurons has been put into context is through its application at the same time as the presentation of a visual stimulus that is expected to activate the same part of visual cortex, while monkeys perform a perceptual task involving the visual stimulus. The expectation is that artificially introduced and visually induced sensory signals will integrate, which could provide a more natural, task-relevant brain activity pattern for investigating the contribution of visual neurons to perception.

### (a) Combined electrical and visual stimulation reveals a perceptual shift

**Figure 3.**
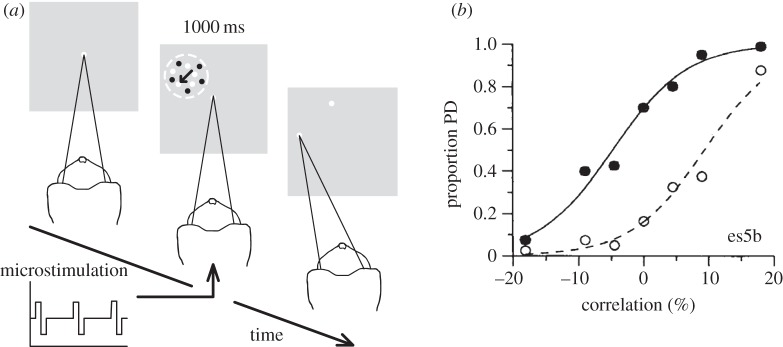
(*a*) Illustration of a trial for the visual motion direction discrimination task with combined electrical microstimulation, developed by Salzman *et al.* [60,62]. After the animal acquired the fixation point, the visual stimulus, a random dot kinetogram, was presented within the receptive field (white dashed circle) of the selected V5/MT site. Electrical microstimulation of the V5/MT site was applied in a randomly selected 50% of trials during visual stimulus presentation. The black arrow within the receptive field indicates the preferred (PREF) motion direction of the stimulated V5/MT site; the opposite direction is the non-preferred (NULL) direction. Upon visual stimulus offset, the animal made an eye movement to the visual target corresponding to its perceptual decision about motion direction. In both microstimulated and non-stimulated trials, animals received a fluid reward if they made a correct choice with respect to the visual stimulus. (*b*) An example of the effect of V5/MT microstimulation on perceptual decisions in the motion task, taken with permission from Salzman *et al.* [60]. The proportion of PREF direction (PD) choices made by the animal was plotted against the percentage of visual stimulus dots moving in the PREF direction (positive correlation) or in the NULL direction (negative correlation). Black circles (smooth line) indicate choices on microstimulated trials; white circles (dashed line) indicate choices on not electrically stimulated trials. For a given motion correlation strength, the proportion of choices towards the PREF direction was greater on trials in which microstimulation was applied, as expected under the hypothesis that the activation of direction-selective V5/MT neurons causally contributes to perception of visual motion.

Salzman *et al.* [60,62] devised a causal experimental approach that overcame many of the previously discussed limitations. They combined systematic neuronal recordings and visual stimulus presentation with quantitative measurements of the perceptual effect of electrical microstimulation. Rhesus monkeys performed a motion task in which they discriminated the overall direction of motion of a random dot kinetogram. They reported decisions with an eye movement (saccade) to a corresponding response target in order to receive a fluid reward if correct (figure 3*a*). Clusters of neurons in visual area V5/MT increase their firing rates for a ‘preferred’ direction of visual motion, and decrease firing for the opposite (‘null’) direction [63,64]. Salzman *et al*. [62] characterized the receptive field location and direction preference of a V5/MT site, and matched the random dot stimulus to the site's receptive field. The percentage of dots moving in the preferred direction was varied systematically from trial to trial. Electrical microstimulation (10 µA current) was applied to the V5/MT site during visual stimulus presentation in a randomly selected half of the trials. Animals were significantly more likely to report motion in the preferred direction on microstimulated trials compared with non-stimulated trials, demonstrating that activation of the V5/MT site biased motion perception towards the neurons' direction preference [62]. This bias was evident in a consistent shift of the whole psychometric function, i.e. it was evident for stimuli that were harder to discriminate and stimuli that were easier to discriminate (figure 3*b*). Increasing the stimulation current to 80 µA eliminated the directional bias and impaired overall performance, indicating that this current level introduced noise into the cortical area and may have spread beyond the selected V5/MT site. Changing the electrode position on the order of 100 µm also abolished the bias [65]. This suggests that electrical microstimulation in area V5/MT primarily activates small, localized populations of neurons with similar tuning preference, perhaps on the scale of a cortical column [64]. These experiments established the causal role of V5/MT neurons in the perception of visual motion by tightly and quantifiably linking the activation of specific neuronal representations to perception.

Applying this experimental approach, others have quantitatively demonstrated the causal contribution of different cortical areas to visual perception as predicted from neurons' specific tuning properties (figure 1*c*). For instance, neurons in the medial superior temporal (MST) area are also selective for motion direction, but with larger receptive fields than in V5/MT, and some are selective for concentric motion [66–69]. Electrical stimulation of MST sites causally biased perception of both motion direction and heading direction (optic flow) according to the direction preference of the stimulation site [70–72]. Primates use binocular disparities to discriminate visual stereoscopic depth, and many visual cortical areas contain neurons that are tuned to binocular disparity [73,74]. Electrical microstimulation of binocular disparity-tuned neurons in area V5/MT biased stereoscopic depth judgements towards the disparity preference at the stimulation site in a coarse depth discrimination task, whereas stimulating area V4 neurons biased fine judgements of depth between centre and surround, demonstrating a causal contribution of these cortical regions to stereoscopic depth perception but for different stimulus configurations [74–76]. The inferotemporal cortex (visual area IT) contains neurons selective for faces and for specific three-dimensional structures. Afraz *et al*. [77] trained monkeys to perform a categorization task to judge whether visual images were faces or non-faces. Stimulation of face-selective IT sites strongly biased decisions towards the face category, with the magnitude of the effect depending upon the degree of face selectivity of the site [77]. Similarly, in a three-dimensional structure categorization task (concave versus convex), stimulation of structure-selective IT sites biased monkeys' choices towards the preferred structure of the neurons [78]. Thus, combining electrical and visual stimulation has revealed a direct relationship between neural tuning and visual perception for many extrastriate visual areas in the monkey.

### (b) Relationship between neuronal tuning and microstimulation

In all the experiments described in §3a, visual stimuli and task were carefully matched to neuronal response properties at the recording site. The number of studies cited above might suggest that causal activation of tuned visual cortical neurons always successfully biases perception according to their tuning preferences. But is this true? If yes, then diligently performing causal experiments in each and every visual area becomes less important; if not, causal approaches remain necessary to clarify in which situations neuronal activity actively contributes to perception and behaviour. A stumbling block to answering this question is that negative experimental results (an absence of an effect) are less common in the literature, perhaps because it is generally more difficult to assess the validity of a negative result. So for instance, it is difficult to know whether the absence of significant microstimulation effects in combination with visual stimulation for V1 sites (figure 1*c*) means that it does not work or that nobody has tried it.

There are, however, a small number of published examples of negative findings for causal contribution of tuned neurons, usually in the context of a positive microstimulation effect for another stimulus parameter [72,79,80]. For example, although multi-sensory neurons in dorsal MST (area MSTd) are tuned to both optic flow and vestibular heading direction signals [67–69,81,82], microstimulation of such neurons significantly biased monkeys' heading percepts in an optic flow task, but not a vestibular task [72]. The cortical sites identified in the study had generally weak vestibular tuning, and therefore they may not causally contribute to perception of heading direction.

Uka & DeAngelis [80] microstimulated disparity-selective V5/MT sites as monkeys performed both a ‘coarse’ depth task that involved discrimination of larger binocular stimulus disparities relative to the animal's fixation point (absolute disparity), in the presence of visual noise, and a ‘fine’ task that involved depth discrimination of small changes in the binocular disparity of a patch relative to its immediately surrounding annulus (relative disparity) [80]. Microstimulation of area V5/MT biased depth judgements in the coarse task but not in the fine task, even though disparity-tuning curves of some V5/MT neurons were sufficiently sensitive to account for the animal's performance in the fine task [80]. The authors suggest that the tuning of most V5/MT neurons is for absolute rather than relative disparity, regardless of tuning sensitivity (at least in this spatial configuration—see [83]), and that this may be the reason that V5/MT sites they stimulated did not contribute to depth judgements in the fine task. Indeed, many neurons in area V4 encode relative disparity between adjacent surfaces [84], and microstimulation of V4 sites has been shown to bias depth judgements in a fine disparity task [76]. Hence, causal experiments differentially link representations of binocular disparity in cortical areas V5/MT and V4 to the areas' functional contributions to depth perception dependent on the spatial configuration of the stimulus (see also [74]).

Other aspects of the neurons' receptive field, not immediately task-relevant, may also affect their functional contribution to visual perception. One study indicated that only microstimulation of direction-selective V5/MT sites with an antagonistic receptive field surround, but not those without, caused a shift in monkeys' visual pursuit movements towards the preferred direction of the stimulated neurons [85]. This could be due to the importance of antagonistic surrounds in V5/MT to the segregation of object versus background motion. In another study, DeAngelis & Newsome [79] microstimulated motion direction-selective sites in area V5/MT and also measured the stimulated sites' binocular disparity tuning, even though the task itself involved motion direction discrimination only. They found that for two of three monkeys, microstimulation of V5/MT sites that were not tuned to binocular disparity produced the largest bias in direction judgements, while stimulation of direction- *and* disparity-selective sites had little or no effect even when the random dot kinetogram was presented at the cortical sites' preferred disparity [79]. But, when causal activation of a disparity-selective site did influence direction judgements, the effect was strongest when the stimulus was presented in the preferred depth plane. Therefore, even if visual neurons are tuned to a task-relevant stimulus dimension, they may not necessarily contribute to behavioural responses; instead, their perceptual contribution may depend on tuning to other visual features that seem task-irrelevant. Causal experimental approaches should probe more deeply how context affects the contribution of specific visual neuronal representations to behaviour.

### (c) From simple features to visual objects

**Figure 4.**
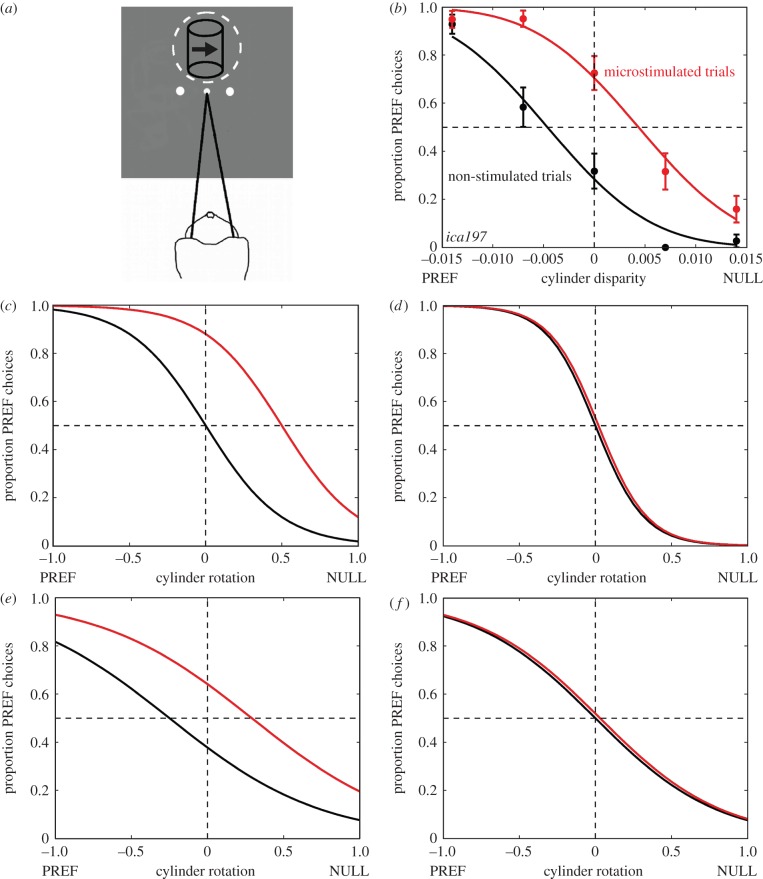
Effect of intracortical microstimulation on judgements about a visual stimulus. Experimental data and simulations of psychometric functions illustrate different microstimulation effects and strategies that may occur. (*a*) Illustration of the visual cylinder task, in which monkeys discriminated the direction of rotation of a transparent structure-from-motion cylinder presented in the receptive field (white dashed circle) of microstimulation sites in extrastriate visual area V5/MT. The direction of rotation was defined by separating front and back surfaces with binocular disparity. The animal indicated its perceptual choice with an eye movement to one of two targets, located at opposite sides of the fixation point. Animals were rewarded for a correct choice with respect to the visual stimulus. (*b*) Gaussian psychometric functions (PMFs) fitted to experimental data from Krug *et al*. [88] with microstimulation at cortical site *ica197,* which was tuned for a negative cylinder disparity. Electrical microstimulation at this site induced a strong perceptual shift in the PMF in the preferred direction (PREF) of the neurons at the stimulation site in V5/MT. The animal's ‘null bias’ also caused the PMFs to shift towards the null direction (NULL), which is apparent in the non-stimulated trials (red lines: microstimulated trials; black lines: non-stimulated trials). Panels (*c*–*f*) illustrate alternative possible outcomes based on data simulations. (*c*) Simulation of the PMFs that we would expect to see if the null bias were not present. The shift in the PMF due to electrical microstimulation would be the same, but the PMF for non-stimulated trials would pass through 50% at zero disparity. (*d*) Simulation for the hypothetical case where an animal could detect microstimulation trials and apply the null bias on microstimulation trials only. As in the discussed experiments, animals would be rewarded for correctly reporting the visual stimulus only. So we would expect that the perceptual shift due to microstimulation might be all but cancelled out by such a strategy. (*e*) Simulated experiment, in which microstimulation detection training (for microstimulation alone) at a direction- and disparity-selective V5/MT site is followed by microstimulation at the same site during the cylinder task. Expected PMF for the cylinder task is shown if microstimulation detection training simply increased visual discrimination thresholds, as reported in Ni & Maunsell [36], without affecting the integration of electrical and visual stimulation. The PMF flattens as performance accuracy decreases, but the bias effect due to microstimulation remains. (*f*) As for (*e*), but now the microstimulation perceptual shift is cancelled out because having been trained to detect microstimulation, animals might be able to distinguish microstimulation trials and apply the null bias on those trials only.

The question of when information represented by neuronal firing is causally relevant to perception, especially of more complex visual objects, has led us to consider conjoint tuning for neurons representing more than one sensory dimension. For example, neurons in visual area V5/MT are tuned to both the direction of visual motion and binocular depth of a stimulus [63,83,86,87]. In the discrimination task described in the previous paragraph, only one parameter (motion) but not the other (binocular depth) was relevant to the animals' task [79]. However, in natural viewing, judgements about visual objects require a combination of multiple parameters, including direction, colour, depth and speed. Krug *et al.* [88] demonstrated that V5/MT neurons contribute directly to judgements about a rotating structure-from-motion cylinder stimulus that *requires* the joint encoding of both motion and depth [88]. The cylinder was made up of two transparent surfaces of random dots moving in opposite directions, such that assigning dots with opposite motion directions to different visual depth planes defined the direction of rotation [89,90]. Monkeys indicated their choice about cylinder rotation direction with an eye movement (figure 4*a*). Electrical microstimulation of a motion- and depth-tuned V5/MT site biased choices towards the rotation direction represented by the conjoint tuning at that site (figure 4*b*). For example, stimulating a site selective for rightwards motion in the near depth plane boosted the rightward motion signal at the near surface of the cylinder only, resulting in an increased proportion of choices for the corresponding direction of rotation [88]. At another site with selectivity for rightwards motion in conjunction with far disparity, choices were biased in the opposite rotation direction. Since both motion directions and depth planes are simultaneously present in the stimulus, neither motion nor disparity selectivity alone can explain this pattern of stimulation results. Therefore, this causal intervention implicates the conjoint tuning for motion and disparity in V5/MT in shaping the visual percept of a structure-from-motion object.

Krug *et al*. [88] compared the size of the microstimulation bias on perceptual choices in their study to a previous study that investigated the effect of stimulating V5/MT neurons in a planar depth discrimination task with the same stimulating current of 20 µA [75]. This was possible because both studies used similar experimental paradigms and, crucially, the same methods to quantify both the selectivity of neurons for depth at the stimulated sites (Disparity Tuning Index) and the behavioural bias induced by the causal intervention (horizontal shift of the psychometric function). Although strength of disparity tuning was similar for cortical sites in the two studies, there was a systematically larger effect of microstimulation on perceptual judgements in the conjoint structure-from-motion task than in the planar depth task [88]. This suggests the presence of a specialized functional organization in area V5/MT representing the relationship between motion and depth. Furthermore, area V5/MT also contains neurons that are not selective for the conjunction of motion and disparity although they are selective for either one alone. Thus, there may be a smaller pool of neurons available for the structure-from-motion task described here, and microstimulation may drive a greater proportion of these neurons, explaining the greater biasing effect. In order to arrive at a better understanding of how visual cortex supports the perception of visual objects and scenes, we require further investigation into the effects of microstimulation in different cortical areas for the same visual task, and in the same area but with different visual tasks.

### (d) How does microstimulation change visual percepts? Evaluating animals' strategies

Experiments combining visual and electrical stimulation in rhesus monkeys demonstrated a shift in perceptual choices towards the visual parameters represented by the stimulated neurons. Does this show integration of visually evoked and artificially introduced electrical activity? To what extent might animals be aware that they are being microstimulated on some trials? As we have seen in previous sections, animals can learn to detect electrical microstimulation in extrastriate visual cortex when specifically trained and rewarded. Human patients report phosphenes and flashes of light under electrical stimulation of visual cortex, albeit under different experimental and stimulation protocols (see tables 1 and 2). It is conceivable that a flash of light might be noticeable to the animal during microstimulation. So, rather than integrating visual and electrical stimulation, could animals have learned to detect when they were being stimulated and act differently on those trials?

**Table 2.** Summary of electrical microstimulation studies combined with visual stimulation in non-human primates (rhesus macaques). This table provides a comparison of the methods employed in combined electrical microstimulation/visual stimulation studies discussed in this review. Abbreviations used: MU, neuronal multi-unit; RT, reaction time, i.e. microstimulation was presented during the visual stimulus presentation, the duration of which was controlled by the animal's reaction time; MSTd, dorsal MST; IT, inferotemporal area; LIP, lateral intraparietal area; FEF, frontal eye fields. (−/+) represents negative- or anode-leading biphasic stimulation, (+/−) represents positive- or cathode-leading biphasic stimulation. For biphasic stimulation, stimulating current is reported as the zero-to-peak amplitude and pulse duration is reported as the duration of each positive or negative phase.

study	stimulating current, µA	stimulation frequency, Hz	stimulation pattern	pulse duration, ms	stimulation duration, ms	recordings	cortical area	behavioural task
Afraz *et al*. [77]	50	200	biphasic (+/−)	0.2	50	face selectivity	IT	face versus object discrimination
Bisley *et al*. [91]	80	200	biphasic (+/−)	0.2	500	motion direction	V5/MT	match-to-sample motion task
Born *et al*. [85]	40	200	biphasic (+/−)	0.2	RT	motion direction and RF surround	V5/MT	pursuit eye movement to target
Britten & van Wezel [71]	20	200	biphasic	0.2	1000	heading motion direction	MST	heading direction discrimination
Carey *et al*. [92]	30–50	200	biphasic	0.2	300	motion direction	V5/MT	smooth pursuit learning
Celebrini & Newsome [70]	10	200	biphasic (+/−)	0.2	1000	motion direction	MST	motion direction discrimination
DeAngelis *et al*. [75]	20	200	biphasic (+/−)	0.2	1000	binocular disparity	V5/MT	depth discrimination
DeAngelis & Newsome [79]	20	200	biphasic (+/−)	0.2	1000	motion direction and binocular disparity	V5/MT	motion direction discrimination
Ditterich *et al*. [93]	5	200	biphasic (−/+)	0.3	RT	motion direction	V5/MT	motion direction discrimination
Fetsch *et al*. [94]	5, 7.5, 10	200, 250, 333	biphasic (−/+)	0.4	mean 270	motion direction	V5/MT	motion direction discrimination
Groh *et al*. [95]	20, 40, 80	200	biphasic (+/−)	0.2	110–180	motion direction	V5/MT	pursuit eye movement
Gu *et al*. [72]	Gaussian envelope, peak 20	200	biphasic (+/−)	0.2	2000	vestibular and visual heading direction	MSTd	heading direction discrimination
Hanks *et al*. [96]	10–20	200	biphasic (−/+)	0.3	RT	motion direction	V5/MT & LIP	motion direction discrimination
Kawasaki & Sheinberg [97]	30	200	biphasic	0.2	1050 or RT	MU tuning not measured	IT	object classification
Krug *et al*. [88]	20	200	biphasic (+/−)	0.2	2000	motion direction and binocular disparity	V5/MT	structure-from-motion rotation discrimination
Moore & Armstrong [98]	mean 40.7	200	biphasic	0.2	20–50	receptive fields in FEF and V4	FEF	fixation task
Moore & Fallah [99]	mean 23.5	200	biphasic	0.2	100	FEF response field	FEF	luminance change detection
Murasugi *et al*. [65]	2.5–80	12.5–500	biphasic (+/−)	0.2	1000	motion direction selectivity	V5/MT	motion direction discrimination
Nichols & Newsome [100]	10 or 25	200	biphasic (+/−)	0.2	1000 or 1500	motion direction selectivity	V5/MT	motion direction discrimination
Salzman *et al*. [62]	10	200	biphasic	0.2	1000	motion direction selectivity	V5/MT	motion direction discrimination
Salzman *et al*. [60]	10	200 or 500	biphasic (+/−)	0.2	1000	motion direction selectivity	V5/MT	motion direction discrimination
Salzman & Newsome [101]	10	200	biphasic (+/−)	0.2	1000	motion direction selectivity	V5/MT	motion direction discrimination
Shiozaki *et al*. [76]	40	200	biphasic (+/−)	0.2	1500	fine binocular disparity tuning	V4	depth discrimination
Uka & DeAngelis [80]	20 or 40	200	biphasic (+/−)	0.2	1500	binocular disparity tuning	V5/MT	coarse versus fine depth discrimination
Verhoef *et al*. [78]	35	200	biphasic (+/−)	0.2	RT (>100)	3D structure selectivity	IT	3D structure categorization

A number of strands of evidence converge to suggest that this is not the case. In all the studies described above, animals did not receive an incentive to bias their behaviour in the direction predicted from the selectivity of the simulation site. Animals were only rewarded for choices that were correct with respect to the visual stimulus, regardless of whether electrical microstimulation was introduced on a particular trial. Therefore, the only incentive animals had was to ignore the microstimulation signal, because they lost some of the available rewards through being biased by microstimulation. In an in-depth study of the microstimulation effect in V5/MT in the motion discrimination task, Salzman *et al*. [60] characterized a behavioural phenomenon, the ‘null choice bias’, whereby monkeys' choices were apparently biased toward the site's null (non-preferred) motion direction in trials without electrical microstimulation [60] (figure 3*b*; see also figure 4*b* for the cylinder task). This bias was not present in prior psychophysical testing without microstimulation trials. Salzman *et al*. [60] presented the results of several experiments that explain this null bias as a probability-matching behavioural strategy, in which the animal makes a roughly equal number of responses in favour of the preferred and null directions over the course of the experiment to match overall choice proportions to the reward contingencies (similar to when reward probabilities change for different options, see [102,103]). The null choice bias is evident for non-microstimulated trials, because it was applied across all trials to match reward distributions. If animals could tell the difference between stimulated and non-stimulated trials, the optimal strategy would be to apply the null bias to the microstimulation trials only in order to maximize reward (cf. figure 4*b*–*d*), but this has not been observed experimentally. In contrast, the observed pattern of responses shows that animals did not discriminate between microstimulated and non-stimulated trials (figure 3*b* for the motion task and figure 4*b* for the cylinder task).

Furthermore, in these experiments, stimulating currents ranged predominantly between 10 and 40 µA (table 2), lower than detection thresholds found for sites in visual cortex *before* extensive training took place to detect electrical microstimulation [36]. Such training, involving 1000s of trials, was necessary for detection thresholds of microstimulation alone to reach a stable low value (discussed in §2b(ii)). Considering further that detection thresholds did not fully generalize under training across cortical sites in one visual area [36], and that in Salzman *et al*. [62] and similar studies, a new microstimulation site was selected at least once each day, it is unlikely that animals had sufficient training opportunity to reliably detect the amplitude of microstimulation in the extrastriate visual areas tested. Finally, there is a contextual difference between detecting punctate electrical stimulation in an otherwise not specifically stimulated visual region in a pure detection task, and detecting the same activation in a visual area that is specifically activated by a concurrent visual stimulus, such as area V5/MT by the random dot kinetogram. Identifying the artificial stimulation among simultaneous visually evoked neuronal activity may be much more difficult.

Overall, the evidence strongly suggests that animals were not able to detect when they were being microstimulated in experiments combined with visual stimulation. Nevertheless, subjects' potential capacity for perceptual learning to detect microstimulation during visual stimulation is an important question. In order to evaluate the extent to which electrical microstimulation and visual stimulation are comparable and detectable, it would be important to test whether microstimulation detection training (as in [34]) would increase, decrease or have no effect on the microstimulation-induced perceptual bias in a visual discrimination task, and whether it would affect relevant discrimination thresholds (figure 4*e*,*f*). This would also provide further insight into the sites of synaptic changes underlying perceptual learning.

### (e) Current gaps in understanding

Measuring the effect of electrical microstimulation on the performance of well-controlled visual tasks has established the contribution of a number of extrastriate visual areas to visual perception. This technique has extensively characterized the contribution of dorsal stream areas V5/MT and MST to motion and depth perception; a smaller number of studies have linked inferotemporal cortex (area IT) to face and shape perception and area V4 to depth perception. Based on these studies, there are gaps in our ‘causal map’ concerning the specific perceptual contribution of visual areas V1, V2 and V3; moreover, the ventral stream is under-represented overall (figure 1*c*). Considering that area V1 has been a popular site to study the detection of electrical stimulation in animals and humans (figure 1*a*,*b*) and has a well-established columnar organization, it seems surprising that perceptual shift studies have not been carried out in V1 yet. Perhaps electrically elicited phosphenes are too bright to allow any discrimination of visually evoked percepts of, for instance, stimulus orientation.

Studies investigating the causal contribution of colour representations are also notably missing from current literature with non-human primates. This is at odds with human stimulation studies that successfully combined recording and stimulation of colour areas in the temporal lobe [18,20]. Investigating causal contributions of, for example, areas V1 and V4 to colour discrimination in monkeys could provide further evidence for the progression from wavelength selectivity in early cortical areas to colour constancy down the ventral stream [104–106]. These apparent ‘gaps’, if due to failure to elicit significant microstimulation effects in the corresponding areas and tasks, could point to differential interactions between a punctate electrical stimulation source and the cortical architecture (see also §2b(i)).

Finally, there are clear advantages to being able to compare quantitatively the results of electrical stimulation across studies (for example, [88]). Notwithstanding time and other limitations, future microstimulation studies with human patients would benefit from incorporating quantitative measures of discrimination judgements in visual stimulation tasks, in addition to recording subjective reports, in order to better enable comparisons across studies and species.

## 4. Investigating mechanisms of visual cognition

In addition to exploring functional properties of different visual cortical areas and their contribution to perception, vision research also investigates the mechanisms of cognition. Causal approaches, such as electrical microstimulation and pharmacological interventions, play their part in testing different models of these mechanisms. In this section, we briefly review three examples in which microstimulation was used to elucidate neuronal mechanisms for memory, attention and perceptual decision-making (figure 1*d*).

### (a) Cortical mechanisms for working memory and perceptual learning

The approach to combine electrical microstimulation with specific visual stimulus presentation has been extended to study the localization of cortical changes underlying memory and perceptual learning within visual cortex. Bisley *et al.* [91] trained macaque monkeys in a working memory task in which animals had to press one of two buttons to indicate whether the direction of motion of a test stimulus was the same or different from a previously presented sample stimulus [91]. Electrical microstimulation of direction-selective V5/MT sites during the presentation of the sample stimulus biased animals' choices towards those test stimuli whose motion direction matched the neurons' preference. During the delay period, stimulating an opposite motion direction to the sample disrupted performance. Neurons in area V5/MT can therefore provide the directional information for visual working memory where visual motion is involved.

Electrical microstimulation of visual cortex has also been used during perceptual learning paradigms. Kawasaki & Sheinberg [97] paired electrical stimulation of area IT with perceptually ambiguous visual patterns, and showed that the differential activity of electrical stimulation combined with an otherwise identical visual stimulus was sufficient for learning new perceptual classifications. In another study, by Carey *et al*. [92], animals performed a smooth pursuit task in which they learned to predict a precisely timed change in the motion direction of the visual target. When the change in visual target direction was replaced by microstimulation of direction-selective neurons in area V5/MT, animals learned to modulate their eye movements in a manner similar to when instructed by real visual motion. These studies suggest that activation of visual cortical areas can provide a powerful and precisely timed instructive signal for both learning and memory. Further experiments are needed to determine the sites of the underlying synaptic changes.

### (b) Functional connectivity mediating attentional signals in visual cortex

Spatial attention involves the enhancement of visual signals at a location of interest [107–110]. For example, when attention is directed to a preferred stimulus within a V4 neuron's receptive field, firing rates of orientation-tuned V4 neurons were amplified by around 20% [109]. It has been hypothesised that the same circuits that mediate preparation of eye movements (saccades) to a visual target also contribute to covert attention towards a visual location of interest [111]. Moore & Armstrong [98] provided evidence for a direct link between oculomotor control and attention by stimulating sites in the macaque oculomotor area, frontal eye field (FEF), while simultaneously recording from single neurons in visual area V4. Sub-threshold microstimulation of FEF, used in order not to evoke saccades, resulted in an enhancement of visual responses of V4 neurons to a preferred stimulus in a manner comparable with that found in studies of spatial attention [109]. This effect was dependent on the spatial correspondence between FEF saccade response fields and the V4 neuron's receptive field; when non-corresponding sites were stimulated, V4 responses were suppressed. Sub-threshold FEF stimulation also significantly improved animals' performance in a visual dimming detection task: when the visual target was placed inside the stimulated FEF response field, performance was improved in a manner comparable with the effects of spatial attention, but when stimuli were placed outside the response field, performance was impaired [99].

Electrical microstimulation combined with pharmacological intervention was used to investigate the neuropharmacological basis of the enhancement of V4 responses by FEF activation. The FEF neurons' response field was elucidated by microstimulation and then small volumes (0.5–1 µl) of a selective dopamine D1 receptor (D1R) antagonist or D2 receptor (D2R) agonist were delivered to the FEF site. V4 neuronal responses to visual stimuli were altered when D1R activation was manipulated, in a manner similar to attentional effects, but were not affected by the D2R manipulation [112]. The enhancement of V4 signals was restricted to neurons with receptive fields that overlapped with the FEF response field, indicating a potential role for dopaminergic neuromodulation in mediating spatial attention.

Taken together, these experiments provide evidence for an inextricable link between a cortical area that governs oculomotor control and the visual effects of attention, down to the level of neurotransmission (for a wider discussion of oculomotor circuits, see Wurtz [113] in this issue). Combining electrical and pharmacological causal approaches to link prefrontal cortical activity, visual cortical and subcortical activity, and animal behaviour, could elucidate the functional circuits that mediate other top-down processes in visual cognition, such as feature attention [109,114].

### (c) Cortical mechanisms for perceptual decision-making

The function of visual cortex cannot be understood simply by mapping cortical representations of the visual world; we must also understand the mechanisms by which this information is read out to guide behavioural choices. Inserting an artificial signal into cortical representations of sensory evidence allows us to test models of how visual information is utilized by downstream areas to guide behaviour.

Microstimulation detection experiments have been used to investigate mechanisms for the perceptual integration of neural signals in different parts of the same cortical area. Ghose & Maunsell [38] stimulated pairs of V1 sites either singly or jointly, at currents between 1 and 20 µA, while monkeys reported detection of activation at either cortical site. For sites separated by less than 1 mm, animals' detection ability was well described by linear summation of single site current levels. At larger separations, performance was determined by the maximum of the two sites [38], indicating a ‘winner-takes-all’ competition. A limitation of this approach is that brain circuitry may be optimized for integrating naturally evoked patterns of activity but not those with the artificial spatio-temporal properties of electrical microstimulation. Nevertheless, these results indicate that signal integration in V1 is dependent on spatial configuration.

Earlier studies in V5/MT investigated whether read-out mechanisms for motion evidence were best described by a ‘winner-takes-all’ algorithm, in which only the strongest focus of activation informs the decision, or alternatively by ‘vector averaging’, in which all direction-selective V5/MT columns contribute in proportion to their response intensity. Electrical microstimulation of direction-selective V5/MT sites in a visual direction discrimination task that allowed for veridical choices provided evidence that both methods might be used depending on the precise configuration of the task. Mirroring the greater cortical distance of the two stimulation sites in the V1 study [38], for large angular separations between visually and electrically stimulated neurons (greater than 135° of preferred motion direction), or in tasks in which responses must be binned into one of two opposing categories, results were consistent with a ‘winner-takes-all’ mechanism [100,101]. For smaller separations, and in a visual pursuit task, there was clear evidence for vector averaging [95,100] and therefore signal integration.

More recently, electrical microstimulation was used to probe the extent to which the quantitative framework of an accumulation-to-bound model can accurately describe the neuronal mechanisms underlying perceptual decision-making in two-alternative forced-choice tasks [115], such as the direction discrimination task used by Salzman *et al*. [60,62]. In this model, sensory evidence towards each of two competing perceptual choices is represented by firing rates of visual neurons tuned to decision-relevant stimulus parameters, and the subsequent integration of evidence over time is represented by neuronal firing rates in sensorimotor cortical regions, such as area LIP. When firing rates reach a particular decision threshold, the corresponding decision is made [116–118]. Direction-selective sites in area V5/MT, representing sensory evidence, were electrically stimulated while monkeys performed a version of the direction discrimination task in which the duration of motion viewing was controlled by the monkey (reaction time task, [93]). In addition to biasing perceptual choices, as previously reported [60,62], microstimulation also quickened decisions in favour of the stimulated sites' preferred direction and slowed decisions towards the null direction [93]. Microstimulation affected response times even on trials where it did not induce a preferred direction choice. This causal intervention supports the hypothesis that sensory evidence for competing visual interpretations, represented by V5/MT neurons, is accumulated and compared at a subsequent stage.

This framework is also supported by a microstimulation study that demonstrated differential effects of stimulating V5/MT and LIP neurons in the motion task [96]. V5/MT microstimulation had stronger effects on decisions than did LIP microstimulation; moreover, LIP microstimulation has a greater relative effect on reaction times than on choices, compared with V5/MT. In the accumulation-to-bound framework, this is explained by a change in V5/MT firing being integrated as a function of time, and therefore having a substantial, cumulative effect on the decision, whereas the effect of stimulation of LIP is not cumulative [96]. In this way, causal approaches have been instrumental in supporting an accumulation-to-bound model of visual perceptual decision-making.

The perceptual effects of microstimulation itself have also been investigated in the context of this decision-making framework. Monkeys were trained on a variant of the motion task in which they were sometimes allowed to ‘opt out’ of the decision for a small but certain reward, perhaps if their confidence in the correctness of their choice was low [94]. Microstimulation of direction-selective sites in area V5/MT at low currents (5–10 µA) biased perceptual choices towards the preferred direction, but did not reduce overall confidence in the decision. Instead, the effect of microstimulation on decision confidence mimicked a consistent change in the visual motion signal plus a small increase in sensory noise [94]. This demonstrates that artificial manipulation of signals in V5/MT preserves the relationship between accumulated evidence and decision confidence, such that from the perspective of downstream areas, cortical microstimulation is largely equivalent to changes in neural activity produced by a visual stimulus. To directly test the hypothesis that microstimulation affects the drift of the decision variable represented in area LIP in the same manner as equivalent motion energy added to the visual stimulus, it would be necessary to record from neurons in area LIP during microstimulation of area V5/MT when a perceptual decision is taken.

Given that artificial activation of a visual cortical area representing sensory evidence can be integrated almost seamlessly into animals' perceptual decisions about visual stimuli [94], electrical microstimulation could be used to dissect the contribution of different brain processing stages to decision-making. For example, interactions between microstimulation and contextual factors, such as reward, should vary depending on the cortical area in which microstimulation is inserted and the levels at which reward information is represented and integrated into the process. Causal intervention studies investigating the accumulation-to-bound model of perceptual decision making have been largely limited to the motion task and areas V5/MT and LIP (figure 1*d*). Similar experiments using other tasks, such as depth discrimination, or stimulating a different visual cortical area, such as area IT in the context of a face discrimination task, would be necessary to test whether these mechanisms generalize across visual cortex.

## 5. New horizons for causal interference methods

### (a) Optogenetics

Optogenetics is a state-of-the-art causal experimentation method in which light is used to selectively control specific neuronal populations that have been genetically modified to contain light-sensitive proteins [119,120]. These proteins can be coupled to ion transporters or channels, so that illumination of the neurons increases ion movement across the cell membrane and thereby changes cell activity [121]. Optogenetic techniques have been successful in modulating stereotypic behaviour in invertebrates [122–124] (see also Oswald *et al*. [125] in this issue) and driving behavioural responses in rodents (e.g. see [126,127]; see also Saunders *et al*. [128] in this issue). The potential of optogenetics as a method for advancing understanding of visual processing, beyond that of other causal methods such as electrical microstimulation, is based upon its ability to target different groups of neurons that cannot be differentiated by the microelectrode. With optogenetics, photosensitive proteins can be targeted to particular subclasses of neurons, such as GABA-ergic inhibitory neurons or excitatory pyramidal cells, or neurons that send axons to a particular brain structure. This allows selective causal interrogation of the contribution of different cell types to visual processing and behavioural responses, and thereby linking anatomical and functional knowledge about the visual cortex with more precision and specificity than electrical microstimulation. Furthermore, unlike electrical microstimulation, which is generally thought to increase neural activity, optogenetic methods can be used to either activate or silence neurons, depending on the nature of the photosensitive protein [121]. This allows a fine-tuned perturbation of activity that could reveal detailed elements of the neural code.

Optogenetic approaches have already been used to investigate visual processing in rodent models. In one study, activity in visual cortex in mice was inhibited by activating parvalbumin-expressing inhibitory neurons with channelrhodopsin-2 (ChR2), a photosensitive protein linked to a non-specific cation channel. Mice were trained to detect changes in the contrast or orientation of visual stimuli, and their detection thresholds were measured. Suppressing activity in V1 substantially impaired detection thresholds, demonstrating that cortical representations are important for simple visual behaviours in rodents [129]. In another study, mice were trained to report detection of optogenetic activation of excitatory pyramidal cells in area V1 while light pulses were varied in duration and arranged into trains of varying temporal frequency. Detection behaviour was predicted by total spike count, independent of the temporal arrangement, providing evidence for a linear integration mechanism in rodent visual cortex [130].

Although optogenetic techniques have revealed mechanisms of rodent visual cortical processing, translating results from rodent models to primates is not straightforward because of species differences in visual ability, cortical size and the relative importance of vision compared with other senses; these differences are also evident in their different behavioural repertoires and habitats. However, optogenetic approaches in non-human primate models have been slower to develop. Building on an approach that used ChR2-mediated activity in frontal cortex to influence saccade latency [131], one study reported a behavioural response evoked through optogenetic activation of the visual system [132]. Macaques either fixated a central point or made a saccade to a visual target. ChR2 was introduced into area V1 via a viral vector, which was pressure injected. When optical stimulation was applied, the saccade endpoints after fixation point offset were significantly biased towards the receptive field of the optogenetically stimulated neurons. Moreover, saccade latencies were significantly longer when optogenetic stimulation was applied at the same time and in the same receptive field location as a visual target compared with when the visual target was presented alone [132]. Effects on saccades latencies appear comparable with those found in electrical microstimulation experiments in area V1 [133]. This is an important proof of concept that optogenetic techniques can be used to drive behaviour in non-human primates as well as in rodents (see table 3 for a summary of methodological parameters across species).

**Table 3.** Summary of optogenetic studies in visual cortex. This table provides an overview of the methods employed in the recent optogenetic studies of visual cortex discussed in this review. Abbreviations used: AAV, adeno-associated viral vector; ChR2, channelrhodopsin-2.

study	species	transfer particles	cortical area	excitation or inhibition	behavioural task
Gerits *et al*. [131]	rhesus macaque	AAV5-CAG-ChR2-GFP	F5 and FEF	excitation	visually guided saccades
Jazayeri *et al*. [132]	rhesus macaque	rAAV1-SYN1-ChR2(H134R)-mCherry	V1	excitation and inhibition	fixation task and saccade-to-target task
Glickfeld *et al*. [129]	PV-Cre JAX mouse	AAV2/8-DIO-ChR2-mCherry	V1	reversible inhibition (by activation of parvalbumin-expressing neurons)	detection of contrast and orientation change
Histed & Maunsell [130]	Emx1-Cre mouse	AAV2.8-ChR2-mCherry	V1	excitation (by activation of pyramidal neurons)	detection of V1 optogenetic stimulation

A number of technical hurdles currently limit the application of optogenetic techniques in primates compared with other animal models. For instance, it is more difficult to genetically target particular groups of cells whose activation would transmit a coherent signal to downstream brain areas. According to the results from electrical microstimulation detection experiments, the deepest layers of area V1 support the lowest detection thresholds [31,34,35]. However, Jazayeri *et al*. [132] found that in spite of the viral vector having been injected uniformly throughout the cortical depth, channelrhodopsin-2-positive cells were mainly found in layers IVB, with some scattered expression in the deepest layers V and VI [132]. Development of techniques to target particular neuronal cell types and particular layers of visual cortex might be necessary to make the optogenetic technique more effective in primates. In order to generate effective protocols, visual task training also must be matched carefully to the perceptual experience which a particular optogenetic protocol might give rise to. Different microstimulation studies reporting ‘dark’ versus ‘light’ phosphenes indicate that the generated perceptual experience itself requires investigation [23,27,37]. The goal would be to use optogenetics not simply to evoke behavioural responses, but to combine optical stimulation with visual stimulation to further interrogate the contributions of different neurons and activity patterns in visual cortex to the perception of visual attributes such as orientation, motion and colour, as well as to object recognition more generally.

### (b) Visual prosthesis through cortical stimulation

The body of work in humans and animals indicates that area V1 is a good candidate for reliable detection of electrical stimulation. In principle, a prosthesis made of electrodes, linked to a video camera, could be implanted to generate representations of visual information, thereby allowing some extent of sight recovery in blind patients. A prototype visual prosthesis was developed that stimulated six phosphene-inducing V1-surface electrodes in such a way that the letters of the braille alphabet could be read with an accuracy of 73–85% [22]. More recently, an edge-detection processor combined with a television camera and visual prosthesis (64-electrode array implant) allowed a blind individual to navigate between objects in a room [24].

The effects introduced by surface electrical stimulation can be unpredictable and heterogeneous between individuals, and the underlying physiology is not fully understood [43]. With improvements to aseptic chronic implantation techniques, intracortical microelectrodes may provide an even more effective prosthetic approach, targeting specific subregions of cortex and using smaller currents. A chronic (2-year) implantation of an array of 100 penetrating microelectrodes in a macaque resulted in consistent behavioural responses to stimulation across the array, providing evidence for the feasibility of this approach in human patients [134]. Given the development of arrays with ever-larger numbers of electrodes, visual information could be conveyed through increasingly complex patterns of electrical stimulation of visual cortex. As we have seen, work in animals suggests that the interactions between different foci of activation can differ dependent on distance [38], but the type of percept evoked by more complex patterns of stimulation is not well understood. Electrical cortical prostheses may be associated with certain perceptual distortions, as discussed with regard to artificial stimulation of the retina by Fine & Boynton [135]. The capacity of adult primate visual cortex to make perceptual sense of novel stimulation patterns [34,36] and to learn novel perceptual associations [97] suggests that cortical electrical prostheses will one day be a viable option for sight recovery.

## 6. Summary and conclusion

Causal experimental methods have been responsible for important findings in vision research. Focally localized electrical stimulation of human visual cortex, especially in early visual cortical areas, gives rise to distinct visual experiences. Further explorations of effects on perception and learning are required to better understand how specific cortical activations give rise to visual experiences in humans. Studies in which animals have been trained to detect electrical stimulation of their visual cortex reveal principles of cortical coding and plasticity and demonstrate that animals can learn to detect novel patterns of neuronal activity in striate and extrastriate visual areas. This basic research builds a necessary prerequisite to consider the function of visual cortical prostheses.

Electrical microstimulation combined with visual stimulation in forced-choice tasks has successfully been used to demonstrate the contribution of visual cortical neurons to animals' visual perception. Microstimulation quantifiably biases animals' perceptual choices towards the visual features represented by the tuning properties of the activated neurons. Most such studies have focused thus far on a small number of extrastriate visual areas, including V5/MT, MST, IT and V4. The gaps in the monkey's cortical microstimulation map—including V1, V2 and V3—are surprising given that humans and monkeys can detect V1 stimulation alone reliably (figure 1). Relevant experiments may simply not have been attempted thus far. Alternatively, cortical architecture might support different interactions between electrical signals generated by visual input and cortical microstimulation at different cortical sites. The answer to this question will contribute to our understanding of cortical codes for underlying visual experience. Further experiments might consider the neuronal processing of colour, from wavelength selectivity to colour constancy, which might be particularly amenable to such an investigation.

Causal methods have also been used to successfully demonstrate functional connectivity between different brain regions, and increasingly to reveal mechanisms of perceptual decision-making by probing the effect of introducing artificial signals at different levels of the proposed decision-making pathways. In order to reveal the cortical codes for perceptual processes, we require techniques that provide more fine-tuned, distributed patterns of activation and inhibition. Control of the spread of neuronal activation, which is difficult with metal electrodes, will be aided by the on-going development of optogenetic techniques, with promising work on non-human primates in development. Further progress in our understanding of the interactions between artificial stimulation of visual cortical neurons and perception will support visual cortical prosthesis to become a viable option for sight recovery in the future.
